# Prognostic significance of the size and number of lymph nodes on pre and post neoadjuvant chemotherapy CT in patients with pN0 esophageal squamous cell carcinoma: a 5-year follow-up study

**DOI:** 10.18632/oncotarget.18665

**Published:** 2017-06-27

**Authors:** Yong-Kun Chi, Ying Chen, Xiao-Ting Li, Ying-Shi Sun

**Affiliations:** ^1^ Key Laboratory of Carcinogenesis and Translational Research (Ministry of Education), Department of Radiology, Peking University Cancer Hospital & Institute, Beijing 100142, China

**Keywords:** esophageal cancer, pN0, survival, computed tomography, neoadjuvant chemotherapy

## Abstract

The prognosis of patients with esophageal cancer improves by using neoadjuvant chemotherapy (NAC). More patients obtain pathological N0 staging (pN0) after surgery. The heterogeneity of prognosis of these patients poses a great challenge of customizing therapeutic strategies for individual patients. The signs of lymph nodes on both pre and post NAC computer tomography (CT) scan can provide more information for evaluation. Therefore, we investigated a new approach to lymph node (LN)-survival analysis by using pre-/post-NAC CT in pN0 esophageal cancer. 79 patients undergone curative resection after NAC obtained pN0 staging. The long and short axis diameter of maximal lymph node (MaxLN) and LN number on pre-/post-NAC CT scans were recorded and assessed for predicting survival by univariate and multivariate survival analysis. The prognosis of patients with esophageal cancer was correlated with the LN size and number on pre-/post-NAC CT. The LN number on pre-NAC CT and short-axis diameter of MaxLN on post-NAC CT remained the independent predictor of overall survival. By using these two factors as classification criterion, N0b group included patients with LN number>4 on pre-NAC CT or short-axis diameter of MaxLN >7 mm on post-NAC CT and the rest patients were included in N0a group. N0a group had a significantly better overall survival than N0b group (5-year survival rate: 75.2% vs. 32.6%). The size and number of lymph node on pre-/post-NAC CT were reliable and important prognostic factors in patients with pN0 esophageal cancer. This new criterion could distinguish these patients into N0a and N0b, according to different prognosis.

## INTRODUCTION

Recent treatment paradigms of esophageal cancer tend to evolve into a multimodality approach to management including surgical resection and preoperative or definitive chemoradiation therapy [1]. Increasingly, neoadjuvant chemotherapy (NAC) is becoming the neoadjuvant treatment of choice for patients with resectable esophageal cancer [2]. A number of these patients obtain ypN0 after neoadjuvant chemotherapy. Although the N classification is the most important prognostic factor in esophageal cancer because patients without lymph node involvement have a better prognosis than those with nodal involvement [3], we find that these patients presented different survival benefit after NAC downstaging occurrence in clinical follow-up. Thus, we wonder what other factors can be indicators of prognosis in patients with pN0 esophageal cancer. More pathological indicators are not available for pN0 patients except T staging. However, the characteristic of lymph nodes before surgery is revealed on the pre- and post-NAC CT.

The size of pathological lymph nodes is a well-known prognostic factor in esophageal cancer, so nodal size is a criterion for predicting nodal involvement. Unlike the sixth edition of TNM cancer staging manual, the new N classification was determined by the number of metastasis lymph nodes [4]. The change of N staging suggests that number of lymph nodes can be important indicator of prognosis. Furthermore, we demonstrate that CT is an effective tool for measuring the size and counting the total number of lymph nodes [5]. In our prior study, we found that the characteristic of lymph nodes on pretreatment CT was related to the patients’ prognosis in other gastrointestinal cancer [6].

Therefore, we decided to review characteristic of lymph nodes on pre- and post-NCA CT for assessing prognosis of ypN0 patients with esophageal squamous cell carcinoma. Further, we investigate a new approach to CT lymph node (LN)-survival analysis for 5 years follow-up in patients with esophageal carcinomas.

## RESULTS

**Figure 1:**
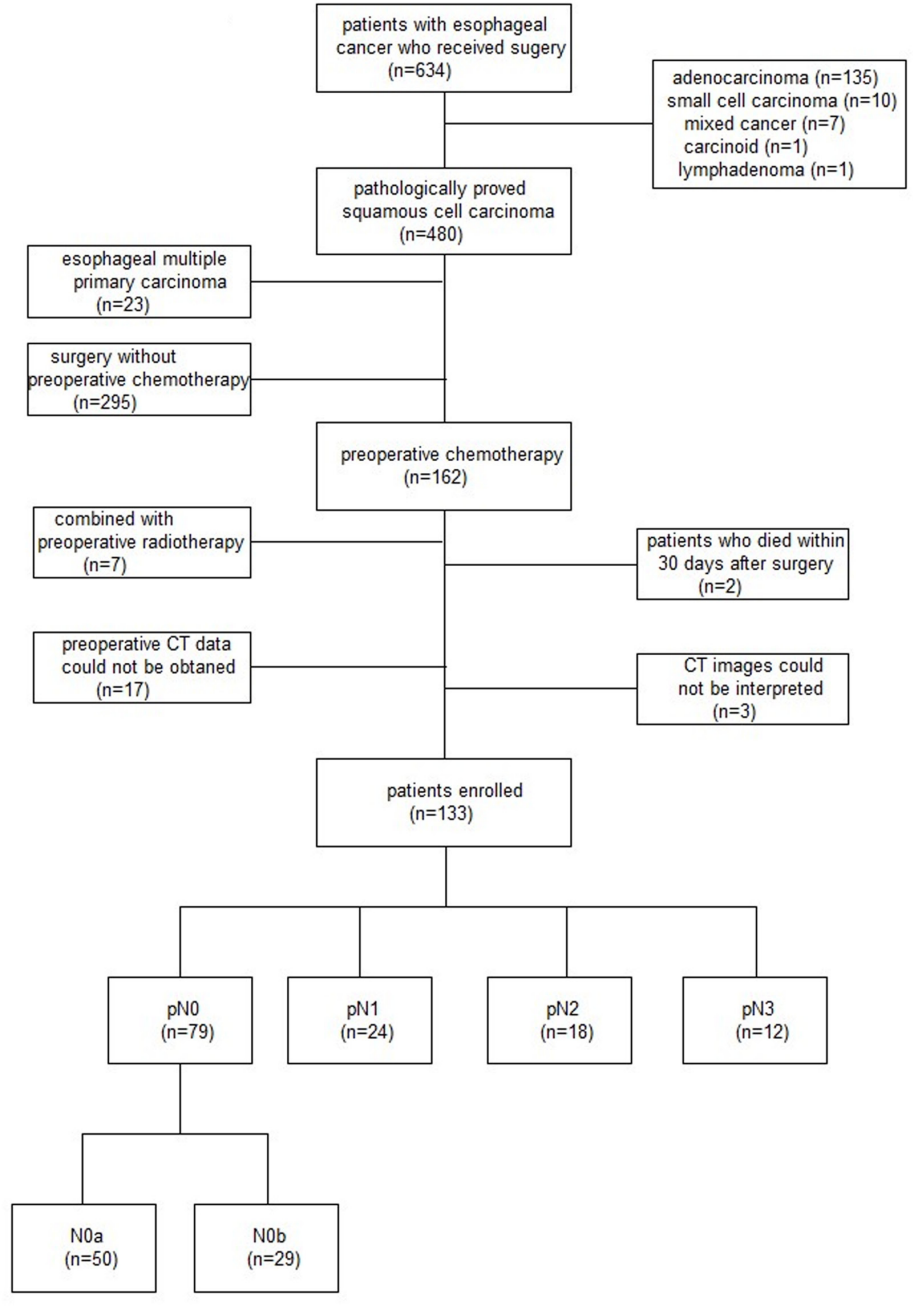
Flow chart of patient enrollment

One hundred and thirty three patients undergone curative resection after neoadjuvant chemotherapy were studied. 79 patients obtaining pN0 staging after surgery were included in the follow up study (Figure 1). There were 59 men and 20 women in the study, with a mean age of 59.87 years (range 42–75). More details of patients’ characteristics and therapeutic regimens were listed in Table 1.

**Table 1:** Summary of patient characteristics and therapeutic regimen

Patient character(n=79)		Description
Age		60±8(42 to 75)
Sex	Male	59(74.7%)
	Female	20(25.3%)
cTNM stages	T3N0M0	9(11.4%)
	T2N1M0	10(12.7%)
	T1N2M0	1(1.3%)
	T3N1M0	21(26.6%)
	T3N2M0	5(6.3%)
	T4aN0M0	3(3.8%)
	T4aN1M0	17(21.5%)
	T4aN2M0	11(13.9%)
	T4aN3M0	2(2.5%)
Pathological differentiation	Well differentiated	20(25.3%)
	Moderately differentiated	41(51.9%)
	Poorly differentiated	18(22.8%)
Neoadjuvant chemotherapy (NAC) scheme	1 cycle	14(17.7%)
	2 cycles	50(63.3%)
	3 cycles	7(8.9%)
	4 cycles	8(10.1%)
Pathological tumor regression grading (TRG)	TRG0	15(19.0%)
	TRG1	22(27.8%)
	TRG2	9(11.4%)
	TRG3	33(41.8%)
Number of lymph nodes analyzed		18±9(6 to 48)

cTNM stages=clinical TNM stages; NAC scheme=neoadjuvant chemotherapy scheme; TRG=tumor regression grading.

The distribution of postoperatively pathological T stages was as follows: 16 patients belonged to pT0, 6 patients to pT1a, 11 patients to pT1b, 19 patients to pT2, 25 patients to pT3, one patient to pT4a and one patient to pT4b.

Sixty-one percent of patients (48 of 79 patients) remained alive, at the last follow-up. The overall 1-year, 3-year and 5-year survival rate were 96.2%, 68.9% and 58.9% for all 79 patients.

### Univariate survival analysis

The results of univariate survival analysis were listed in Table 2. For age and sex of the patients, pathological differentiation grade, tumor location, NAC scheme, pathological tumor regression grading, number of lymph nodes analyzed, cT and cN staging, univariate survival analysis did not show any factor related to the OS and DFS.

**Table 2:** Univariate analysis of prognostic factors according to OS and DFS

Prognostic factor	Number of patients (n=79)	Overall survival			Disease-free survival		
		Rate	95% CI	P value	Rate	95% CI	P value
Age(y)				0.508			0.825
≤59	42	57.6	38.8-76.4		60.6	45.5-75.7	
≥60	37	52.6	35.2-70.0		54.8	37.4-72.2	
Sex				0.191			0.663
Male	59	45.9	25.7-66.1		57.3	43.6-71.0	
Female	20	68.7	47.7-89.7		59.6	37.8-81.4	
Pathological differentiation				0.128			0.133
Well differentiated	20	34.3	10.6-58.0		45	23.2-66.8	
Moderately differentiated	41	61.6	45.3-77-9		62.2	46.1-78.3	
Poorly differentiated	18	71.4	50.2-92.6		77.4	57.8-97.0	
NAC scheme				0.461			0.617
1 cycle	14	49	22.3-75-7		54.4	30.0-81.8	
2 cycles	50	56.1	36.5-75.7		64.9	50.6-79.2	
3 cycles	7	57.1	20.4-93.8		57.1	20.4-93.8	
4 cycles	8	50	15.3-84.7		50	15.3-84.7	
TRG				0.361			0.263
TRG0	15	52.5	26.8-78.2		52.5	26.8-78.2	
TRG1	22	71.8	52.4-91.2		71.8	52.4-91.2	
TRG2	9	33.3	-2.0-68-6		37.5	4.0-71.0	
TRG3	33	65.7	49.2-82.2		64.5	47.4-81.6	
cT				0.273			0.473
cT1	1	-	-		-	-	
cT2	11	63.6	35.2-92.0		63.6	35.2-92.0	
cT3	34	39.1	13.0-65.2		55.2	37.0-73.4	
cT4	33	67	50.1-83.9		68.3	52.0-84.6	
cN				0.810			0.761
cN0	12	60.6	29.8-91.4		54.5	25.1-83.9	
cN1	48	51	32.6-69.4		61.6	46.9-76.3	
cN2	17	56.6	31.7-81.5		58.8	35.5-82.1	
cN3	2	-	-		-	-	
pT				0.03			0.051
pT0	16	55.6	30.9-80.3		55.6	30.9-80.3	
pT1	17	68.2	45.1-91.3		61.4	37.1-85.7	
pT2	19	47	14.7-79.3		61.5	39.2-83.8	
pT3	25	58.4	38.6-78.2		61.2	41.2-81.2	
pT4	2	50	-19.4-119.4		50	-19.4-119.4	
Tumor location				0.157			0.119
Upper 1/3	27	43.1	22.7-63.5		44.3	24.7-63.9	
Middle 1/3	26	43.2	6.16-80.2		60	39.0-81.0	
Lower 1/3	26	69.2	51.4-87.0		68.7	50.7-86.7	
LN Number on pre-NAC CT				0.001			0.002
≤4	34	78.4	62.7-94.1		79.6	64.9-94.3	
>4	45	38.8	21.4-56.2		47.6	32.5-62.7	
Short-axis diameter of MaxLN on pre-NAC CT				0.240			0.125
≤9mm	40	58.0	38.8-77.2		68.1	53.0-83.2	
>9mm	39	52.2	35.0-69.4		53.2	36.3-70.1	
Long-axis diameter of MaxLN on pre-NAC CT				0.050			0.022
≤15mm	40	60.5	40.5-80.5		72.1	57.2-87.0	
>15mm	39	49.9	33.6-66.2		49.9	33.6-66.2	
LN Number on post-NAC CT				0.005			0.014
≤4	39	73.5	58.2-88.8		71.8	56.9-86.7	
>4	40	39.2	21.0-57.4		45.1	28.8-61.4	
Short-axis diameter of MaxLN on post-NAC CT				0.006			0.002
≤7mm	40	64.3	41.2-87.4		75.9	62.2-89.6	
>7mm	39	43.5	27.0-60.0		40.6	24.3-56.9	
Long-axis diameter of MaxLN on post-NAC CT				0.055			0.025
≤12mm	40	59.6	38.6-80.6		70.0	55.1-84.9	
>12mm	39	49.9	33.6-66.2		46.1	29.6-62.6	
LNs analyzed				0.088			0.205
≤17	42	46.2	28.0-64.4		54.8	39.1-70.5	
>17	37	65.3	48.1-82.5		67.6	51.3-83.9	

NAC scheme=neoadjuvant chemotherapy scheme; TRG=tumor regression grading; cT=clinical T stages; cN=clinical N stages; pT=pathological T stages; LN=lymph node; MaxLN=maximal lymph node.

Although pT was found significant for overall survival, only pT4 patients showed statistically poorer OS than other pT stages when we conducted multiple comparisons among pT stages.

Among all the image characteristics, LN number and long diameter of MaxLN on pre-NAC CT were related to the OS and DFS. On post-NAC CT, both size and number of lymph node were demonstrated as prognostic factors significantly.

### Multivariate survival analysis

Multivariate survival analysis, including all statistically significant prognostic factors mentioned in univariate analysis (p value less than or equal to 0.05), was performed to determine the independent prognostic factors for pN0 esophageal squamous cell carcinoma. The pathologic T staging was also included in the analysis. Multivariate analysis by Cox proportional hazard model showed that LN number on pre-NAC CT and LN size on post-NAC CT were most important independent prognostic factors (HR=1.137, 95%CI: 1.041 to 1.240 ; HR=1.083, 95%CI: 1.002 to 1.171) (Table 3). Max LN short diameter was better than long diameter in predicting overall survival.

**Table 3:** Multivariate analysis of prognostic factors

Prognostic factor	Hazard ratio*	P Value
pT	NA	NA
LN Number on pre-NAC CT	1.137(1.041,1.240)	0.004
Long-axis diameter of MaxLN on pre-NAC CT	NA	NA
LN Number on post-NAC CT	NA	NA
Short-axis diameter of MaxLN on post-NAC CT	1.083 (1.002,1.171)	0.044
Long-axis diameter of MaxLN on post-NAC CT	NA	NA

pT=pathological T stages; LN=lymph node; MaxLN=maximal lymph node.

NA=not applicable; hazard ratio not calculated when P≥0.1.

*Numbers in parentheses are 95% confidence intervals.

### Combined criterion for prognosis assessment estimated on CT

We combined these two factors as criterion in order to distinguish the different prognosis for pN0 patients with esophageal squamous cell carcinoma. We chose median values (4 and 7mm) as cutoff points, the patients were divided into two groups. N0b group included patients with LN number>4 on pre-NAC CT or short-axis diameter of MaxLN>7 mm on post-NAC CT and the rest of pN0 patients were included in N0a group (Figure 1). The 1-year, 3-year and 5-year survival rates of the two groups were listed in Table 4. The 5-year survival rate was 75.2% and 32.6% in two group patients respectively, and difference of overall survival time was statistically significant (P<0.001).

**Table 4:** Survival rates of different groups according to combined criteria

Group	N0a	N0b
N	50	29
Mean survival (months)	95.07	54.30
1 year survival rate(%)	100.0	89.7
Standard error	-	5.7
3 year survival rate(%)	85.5	41.4
Standard error	5.1	9.1
5 year survival rate(%)	75.2	32.6
Standard error	6.6	9.1
P value	<0.001	

N0a group: LN number≦4 on pre-NAC CT and short-axis diameter of MaxLN≦7 mm on post-NAC CT.

N0b group: LN number>4 on pre-NAC CT or short-axis diameter of MaxLN>7 mm on post-NAC CT.

**Figure 2:**
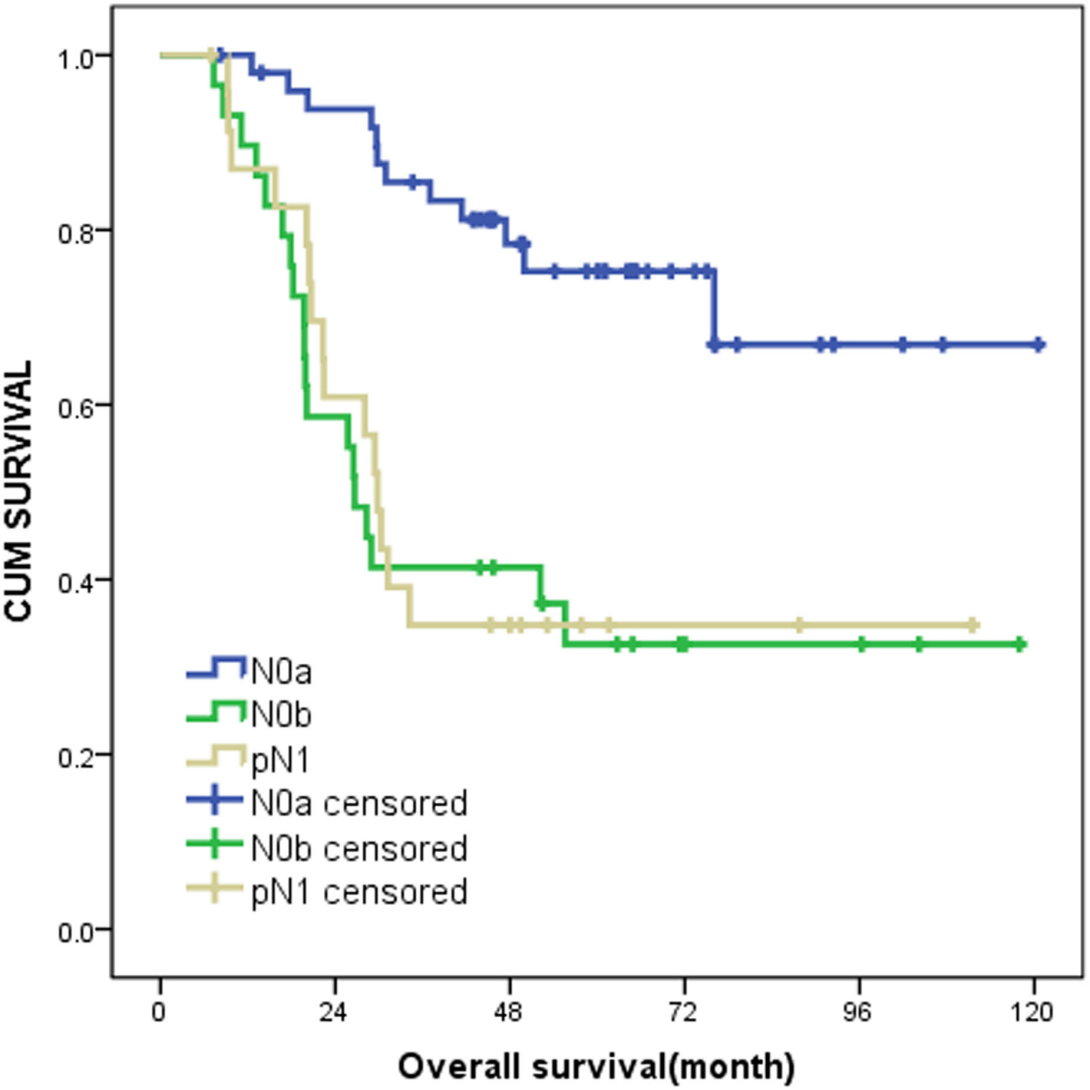
Kaplan-Meier curves of N0a, N0b and pN1 on survival outcomes (N0a group:LN number≦4 on pre-NAC CT and short-axis diameter of MaxLN≦7 mm on post-NAC CT. N0b group:LN number>4 on pre-NAC CT or short-axis diameter of MaxLN>7 mm on post-NAC CT.).

**Figure 3:**
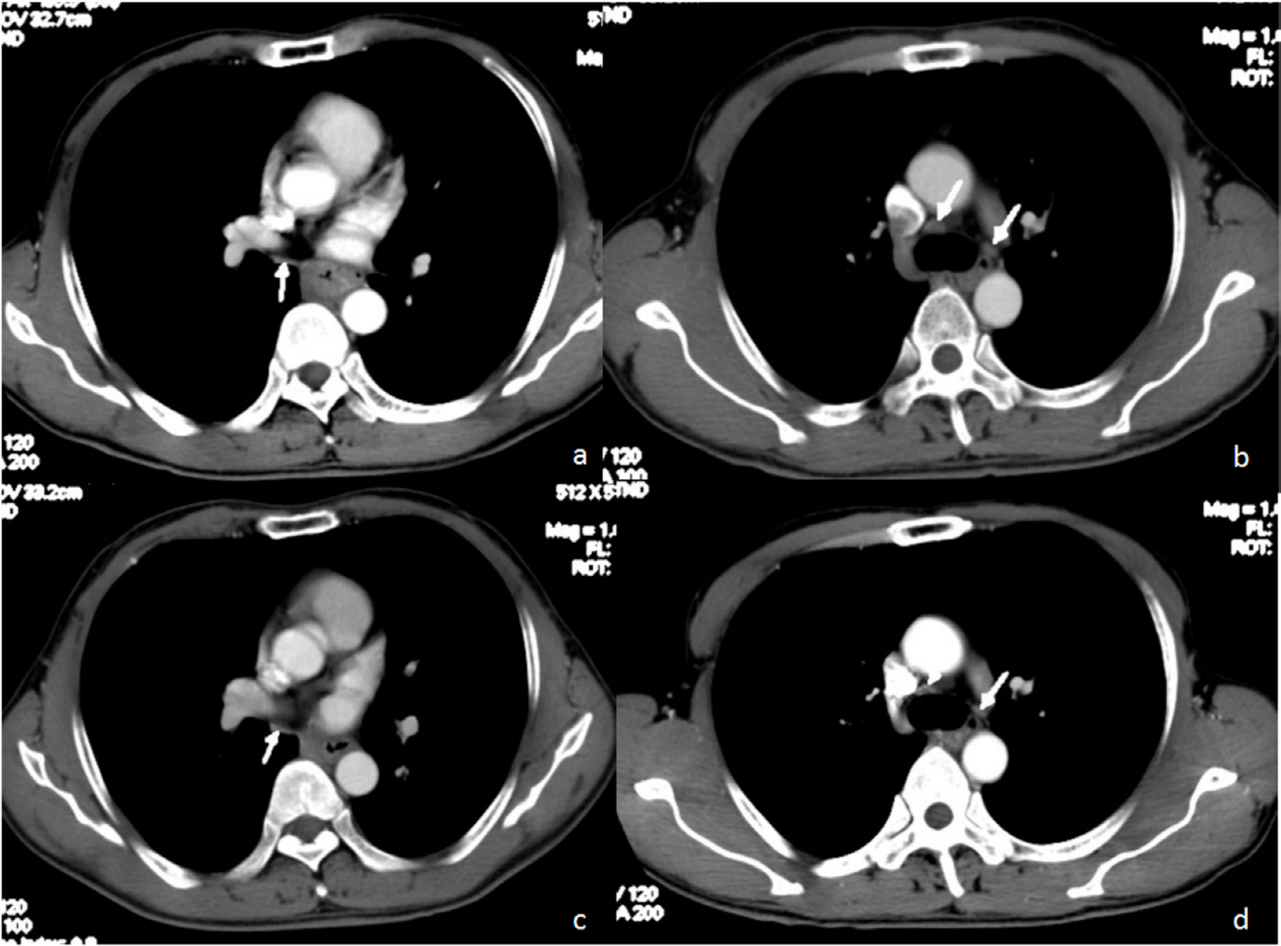
58 year-old man with good prognosis of esophageal squamous cell carcinoma Pre-neoadjuvant chemotherapy CT **(a, b)** showed MaxLN (arrow): 16x5mm, the number of LN (arrow) was 3. Post-neoadjuvant chemotherapy CT **(c, d)** showed MaxLN(arrow):13x5mm, the number of LN (arrow) was 3. OS=70.1 months, DFS=70.1 months.

**Figure 4:**
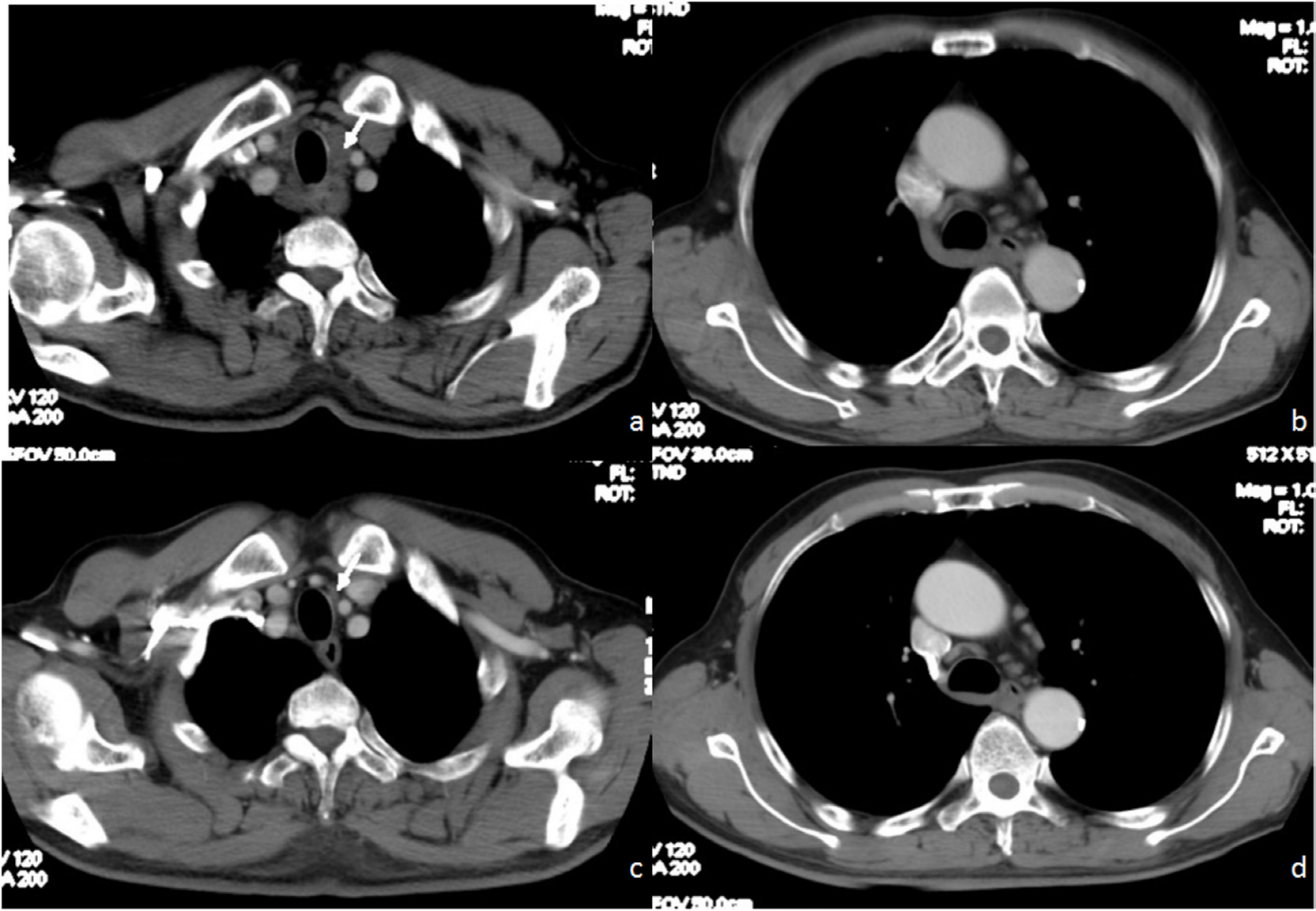
66 year-old man with poor prognosis of esophageal squamous cell carcinoma Pre-neoadjuvant chemotherapy CT **(a, b)** showed MaxLN (arrow): 24x13 mm, the number of LN was 10. Post-neoadjuvant chemotherapy CT **(c, d)** showed MaxLN (arrow):10x6mm, the number of LN was 9. OS=7.3 months, DFS=5.6 months.

Patients in pN0a group (n=50, median survival time not reached) survived longer (Figure 2). The median survival time of remaining patients in pN0b group (n=29) was only 43 months. Moreover, this indicator derived from two preoperative CT examinations provided satisfactory predictability for prognosis. See Figure 3, Figure 4.

## DISCUSSION

### Different influence between N0a and N0b

Esophageal cancer staging is changing gradually and has undergone several revisions with the emergence of new prognostic factors. The post-operative pathological stage is widely accepted to be the best prognostic factor. But the prognosis of patients with pN0 staging was not discussed in previous study. According to the data of this study, none of cT, cN and pT staging was very useful prognostic predictor for these patients. Thus, it suggested that more factors should be considered for predicting overall survival as we could not obtain accurate prediction for OS in pN0 patients. In clinical routine follow-up data, some pN0 patients appeared new metastatic lymph nodes after the surgery. The patients’ survival seemed different within all pN0 patients. Screen out this worse prognostic part of pN0 patients may change the treatment plan.

The results of our study suggested that the size and number of lymph nodes on pre- and post-NAC CT may help us to stratify esophageal squamous cell carcinoma patients with pN0 stage into prognostically different groups.

According to the result of survival analysis, we combined the best two factors into a new criterion, which could distinguish N0a and N0b groups. Because of low accuracy of CT for estimating lymph node metastasis [7], some patients without lymph node involvement in N0a group may be overstaging by pre-treatment CT. So, these patients had better prognosis than patients with extensive lymph node metastasis before treatment. But this factor did not influence all the patients in N0a group. In a word, esophageal cancer with better biological behavior indicated the patients had better prognosis. Our findings confirmed the correlation between severity of LN infiltration and characteristics of lymph node on CT, which was selected as one of the biomarkers. On the other hand, some hazards of lymphadenopathy recurrence were involved in N0b group, which made their survival time similar to patients with pN1 staging. For example, the micro-metastasis of lymph node mentioned in recent pathological literatures may be an important hazard [8]. However, normal-sized lymph nodes which contain microscopic metastatic foci cannot be differentiated from nonmetastatic lymph nodes at CT and can lead to understaging [9].

We also compared the OS of pN0b patients with that of pN1 patients in the same study population (Figure 2). OS of these two groups were not different statistically (p=0.801). Thus, the patients in N0b group may need more aggressive treatment after surgery. We thought that the pre- and post-NAC CT imaging could influence treatment strategies of patients with esophageal squamous cell carcinoma, especially for patients with pN0 staging. The management principle of pN1 patients might be fit for pN0b stage patients.

### LN size on CT scan as independent prognostic factor

The size of lymph node is always a hot issue in cancer studies. Most studies of gastrointestinal tumors consider nodal size as a criterion for predicting nodal involvement. Lymph nodes of 10 mm or greater on CT have been defined as metastasis in esophageal cancer [10, 11].

For tumor patient, lymph node enlargement is an important feature, which is usually detected on CT examination. The diameter of largest mediastinal lymph nodes is about 12mm in healthy population [12]. In contrast, the largest LN diameter detected on post-NAC CT was 22mm of the pN0 esophageal squamous cell carcinoma patients in this study. It demonstrated that the lymph node enlargement was an important change in cancer patients. So, we inferred that the size of lymph node on CT may reflect the severity of esophageal cancer.

We noticed that the short-axis diameter of the largest lymph node on post-NAC CT was one of the indicators of patient survival. In our previous study, the independent prognostic role of LN size was also proved in patients with other gastroenteric cancer [6].

In clinical oncology, lymph node status is one of the most common findings associated with the biological behavior of cancer. LN size remains the strongest independent predictor of survival in patients with esophageal squamous cancer [13]. Larger lymph nodes are more easily to be seen in patients with more aggressive cancer. Thus, the size of lymph nodes on CT scan can reflect the disease progression and patient survival.

### LN number on CT as another predictor of survival

Some pathological studies demonstrate that the number of metastatic lymph nodes is a significant independent survival indicator in esophageal cancer [14, 15]. The AJCC and UICC seventh edition of the staging manual for cancer in the esophagus and esophagogastric junction describes the number of cancer-positive nodes. Several studies have shown that the pN classification by number of LNs is superior to the previous pN classification by the location of the LNs [16–18].

These articles prompted us that LN number on CT could be a prognostic marker, in addition to LN size, in patients with esophageal cancer. In our study, we demonstrate that nodal numbers obtained from pre-NAC CT can be added to the list of prognostic factors for patient survival. In our previous studies, we also discovered that the combined evaluation of these two factors yields a better prognostic prediction than any other factor.

Not all the lymph nodes detected on CT were metastasis in this study. But the pathologic diagnosis of pretreatment lymph node was not available. Although all the patients were diagnosed as pN0 staging after surgery, the number of lymph nodes on post-NAC CT was different obviously. The patients with more lymph nodes on CT images presented poor survival in all pN0 patients. So, it suggests that the number of lymph nodes detected on CT is an indirect feature of the aggressiveness of the tumor. More lymph nodes on CT scans suggest a more adverse prognosis.

### Clinical consequences

Patients with locally advanced esophageal squamous cell carcinoma can benefit from neoadjuvant chemotherapy (NAC) or neoadjuvant chemo-radiation therapy (NCRT). The data from FFCD9901 study [19] suggested NCRT increased incidences of complication and mortality. Many surgeons from China and Japan prefer to choose NAC in treating esophageal squamous cell carcinoma. However most scholars in European and US guessed the preoperative chemoradiotherapy proposed by the CROSS trial was the better preoperative combinational plan [20–21]. In our study, the patients of N0b group did not benefit satisfactorily from NAC alone. So the NCRT plan maybe more effective for these patients.

The characteristics of tumor and lymph node on CT scan can be used to observe the biological behavior of esophageal cancer. It is also revealed in patients with pN0 staging. In this study, our results confirmed that the measurement of size and number of lymph nodes on pre-/post-NAC CT could divide the patients into pN0a and pN0b groups with different survival. CT imaging features would provide important guidance and clues for the prediction of pN0 patients’ prognosis. A part of pN0 patients may benefit from the new staging method by CT imaging.

### LIMITATIONS

First, this is a single center study with a small sample. Second, most of patients didn’t receive preoperative PET-CT, thus we could not use the result of PET-CT as contrast. Third, we didn’t conduct stratification analysis among different pT stages due to the limited sample size.

In conclusion, the size and number of lymph node measured on pre-/post-NAC is a reliable and important prognostic factor in patients with pN0 esophageal squamous cell carcinoma. Therefore, this new criterion can distinguish the pN0 patients into N0a and N0b, according to the different prognosis. It also makes the LN size and number feasible to assess the extent of malignancy, determine prognosis, and aid in the selection of treatment.

## MATERIALS AND METHODS

### Patients

A total of 79 consecutive patients were retrospectively enrolled in this study (Figure 1), who had pathologically proven esophageal squamous cell carcinoma with pN0 staging from November 2005 to December 2011. All the patients were collected from the prospective database of Peking University Cancer Hospital for esophageal cancer.

All the patients were administered pre-NAC contrast enhancement chest CT at our institution as routine staging examinations and then received chemotherapy followed by surgical R0 resection. Clinical staging was obtained mainly using enhanced CT images since few patients received endoscopic esophageal ultrasonography or PET/CT. According to the 7th Edition of the UICC-AJCC TNM Classification for Esophageal Cancer [4], patients in this study were all classified as > cT2 and/or cN+. The post-NAC CT was performed to evaluate the response to neoadjuvant therapies. The pathological staging was performed according to the 7th Edition of the UICC-AJCC TNM Classification for Esophageal Cancer [4]. The distribution of cTNM staging, differentiation grade, pathological tumor regression grading (TRG) and number of removed lymph nodes were all listed on Table 1.

Patients were excluded if: a) other pathological types of esophageal cancer than squamous cell carcinoma; b) they underwent other preoperative therapies simultaneously; c) they had history of other malignancy or esophageal multiple primary carcinoma; d) they received R1 or R2 resection; e) they died within 30 days after surgery; or f) enhanced CT images before and after preoperative chemotherapy could not be obtained or interpreted.

In addition, to protect patient privacy, we removed all identifiers from our records at the completion of analyses. The retrospective investigation project has been examined and certified by Ethics Committee of Beijing Cancer Hospital with waiver of the informed consent.

### Imaging techniques

All patients received enhanced CT scanning within one week before chemotherapy and within one week before surgery. CT examinations were performed with a 64 row helical CT scanner (General Electrical Medical Systems, Milwaukee, WI, Lightspeed VCT). Scans of the chest were performed in the cranio-caudal direction starting from the neck to the renal hilum level. The scans were started 55 s after intravenous injection of non-ionic contrast material (1.5 ml/kg body weight; Omnipaque 300, GE Healthcare) at a rate of 3 ml/s by a high pressure injector via the antecubital vein. The following scan parameters were used: tube peak voltage 120-140 KV, tube current 300 mAs, collimation thickness 1.25 mm, helical pitch 1.5:1. The original data was post-processed at Advantage Workstation 4.2 Image Workstation (General Electrical Medical Systems, Milwaukee, WI), and the coronal and sagittal images were reconstructed using multiplanar reconstruction (MPR) (thickness 0.625 mm).

CT images were analyzed on a PACS station by two independent radiologists who were blinded to clinical and histopathologic information. The short and long diameters of the largest lymph node were measured on CT scans and were considered as the LN size. The total number of all the visible lymph nodes on CT was considered as the LN number. Any nodule larger than 2 mm was deemed as a lymph node. The average of the two radiologists’ results was used in analysis for short and long diameters. The interpretation of the total number was resolved by consensus if there was discrepancy.

### Neoadjuvant chemotherapy and surgery

Platinum-based 2-drug combination, mainly paclitaxel (175 mg/m2, iv, d1 Q21) and cisplatin (25 mg/m2iv, d1-3 Q21) at a 97% (129/133) proportion. The other 4 cases used nedaplatin (80 mg/m2 of body surface area) combined with paclitaxel. Approximately 1-4 neoadjuvant chemotherapy cycles were administered before surgery. Among them, 23, 90, 9, and 11 cases received 1, 2, 3, and 4 cycles, respectively. Surgery was 3-6 weeks after neoadjuvant chemotherapy.

All 133 subjects (pN0 & pN+) underwent radical surgery for ESCC, 114 underwent transthoracic esophagectomy, and 19 underwent transhiatal esophagectomy. Among 114 transthoracic cases, 99 underwent modified Mckeown operation, 10 underwent modified Ivor-Lewis operation, and 5 received trans-left thorax operation (modified Sweet). 111 cases underwent 2-field lymphadenectomy, and 3 underwent 3-field. The number of lymph nodes analyzed was more than six for each patient (Table 1).

### Follow-up

The follow-up consisted of outpatient interviews at 3 month intervals for 2 years, then at 6 month intervals for 3 years, and finally at 12 month intervals until death. Disease-free survival (DFS) was measured from the pre-NAC CT examination date until progression at any site, and patients alive and disease free were censored at the last follow-up. Overall survival (OS) was measured from the pre-NAC CT examination date until esophageal cancer-specific death, and patients alive or dead from other causes were censored at the last follow-up. The follow-up was conducted until June in 2015. The median follow-up time was 64.1 months, ranged from 7.3 to 121 months.

### Statistical analysis

The following parameters from clinical pathological information and pre-/post-treatment CT were chosen as parameters for survival analysis: patient age (≤59 vs. ≥60 years), sex (male vs. female), tumor location (upper middle and lower), pT staging, LN number on pre-/post-treatment CT, short-axis and long-axis diameter of MaxLN on pre-/post-treatment CT images.

The overall survival rate of the different groups as well as the median survival time and survival curves were achieved by using the Kaplan-Meier method. Overall differences in the survival curves were analyzed with the log-rank test.

The multivariate Cox proportional hazards model was used to adjust for the influence of prognostic factors. All statistical analyses and graphs were performed by using the Statistical Package for Social Sciences Program, version 18.0 (SPSS, Chicago, IL). For all analyses, *P* values less than .05 was considered to denote a significant difference.

## Abbreviations

cT: clinical T staging
pT: pathological T staging
cN: clinical N staging
pN: pathological N staging
LN: lymph node
MaxLN: maximal lymph node
NAC: neoadjuvant chemotherapy
CT: computer tomography
TRG: pathological tumor regression grading
MPR: multiplanar reconstruction
DFS: disease-free survival
OS: overall survival
NCRT: neoadjuvant chemo-radiation therapy
